# The effects of aqueous and ethanolic extracts of Rheum ribes on insulin-resistance and apolipoproteins in patients with type 2 diabetes mellitus: a randomized controlled trial

**DOI:** 10.1186/s12906-023-03878-0

**Published:** 2023-02-14

**Authors:** Atieh Ghafouri, Sahar Jafari Karegar, Ghazaleh Hajiluian, Sharieh Hosseini, Shahrzad Shidfar, Mohammad Kamalinejad, Agha Fatemeh Hosseini, Iraj Heydari, Farzad Shidfar

**Affiliations:** 1 Department of Nutrition, School of Public Health, Iran University of Medical Sciences, Tehran, Iran; 2 Student Research Committee, Faculty of Public Health Branch, Iran University of Medical Sciences, Tehran, Iran; 3 Department of Chemistry, Faculty of Pharmaceutical Chemistry, Tehran Medical Sciences, Islamic Azad University, Tehran, Iran; 4 Worcester Memorial Hospital, University of Massachusetts, Worcester, MA USA; 5 School of Pharmacy, Shahid Beheshti University of Medical Sciences, Tehran, Iran; 6 Department of Statistics, School of Public Health, Iran University of Medical Sciences, Tehran, Iran; 7 Institute of Endocrinology and Metabolism, Iran University of Medical Sciences, Tehran, Iran; 8 Research Center for Prevention of Cardiovascular Diseases, Institute of Endocrinology and Metabolism, Iran University of Medical Sciences, Tehran, Iran

**Keywords:** Aqueous extract, Ethanolic extract, *Rheum ribes*, Diabetes mellitus, Apolipoproteins

## Abstract

**Background and aim:**

Previous studies have shown that *Rheum ribes* (*R. ribes*) could be effective in controlling the blood glucose levels. This study was conducted to determine the effects of *R. ribes* supplementation on glycemic indices and apolipoproteins in patients with type 2 diabetes mellitus (T2DM).

**Methods:**

In the present randomized double-blind controlled trial, 60 type 2 diabetic patients aged 30–60 years with a body mass index (BMI) of 20–30 kg/m^2^ and hemoglobin A1c (HbA1c) of 6–8% were enrolled. Patients were randomly assigned to receive 450 mg of aqueous *R. ribes* extract (AG), 450 mg of ethanolic *R. ribes* extract (EG), or placebo (PG) three times daily for 6 weeks. At the baseline and at the end of the study, blood glucose levels, homeostatic model assessment of insulin resistance (HOMA-IR) and the homeostatic model assessment of β-cell dysfunction (HOMA-B), as well as apolipoprotein A-I (ApoA1) and apolipoprotein B (ApoB) were measured.

**Results:**

There was a significant decrease in the serum levels of insulin in AG and EG groups (*P* = 0.003 and *P* = 0.001, respectively), HOMA-IR (*P* = 0.01 and *P* = 0.001, respectively), HOMA-B (*P* = 0.002 and *P* = 0.001, respectively), ApoB (*P* = 0.006 and *P* = 0.03, respectively), ApoB/ApoA1 ratio (*P* = 0.016 and *P* = 0.04, respectively). However, a significant increase in ApoA1 (*P* = 0.08 and *P* = 0.05, respectively) with no significant changes in blood glucose, at the end of study compared to beginning values, were observed. None of the variables showed a significant change in PG. At the end of the study; while there were significant differences in insulin (*P* = 0.04), HOMA-IR (*P* = 0.03), HOMA-B (*P* = 0.01), ApoB (*P* = 0.02), and ApoB/ApoA1 ratio (*P* = 0.03) among the groups but ApoA1 had no significant change.

**Conclusion:**

Consumption of *R. ribes* intake could have beneficial effects on insulin resistance and apolipoproteins in type 2 diabetic patients. (Registered at en.irct.ir, identification number: IRCT201410142709N31).

## Background

Diabetes mellitus is a long-term metabolic disorder characterized by hyperglycemia due to impaired of insulin secretion, insulin function or both. Type 2 diabetes mellitus (T2DM) is a form of diabetes that affects 95% of diabetic patients [1]. The signs and symptoms of this disease are increased serum glucose levels, decreased peripheral glucose uptake due to impaired insulin secretion, and peripheral insulin resistance [2]. According to a report by the International Diabetes Federation (IDF), there are 536.6 million type 2 diabetics worldwide in 2021 and this number is expected to increase to 783.2 million by 2045 [3].

In the last decade, the prevalence of T2DM has increased. T2DM is an important risk factor for the development of cardiovascular diseases such as coronary vascular disease [4] or congestive heart failure [5], chronic kidney disease [6], and also nonhealing wounds [7], that occur because of hyperglycemia. Diabetes is associated with enormous costs to society, such that all countries worldwide spend 5–13% of their costs on the treatment of diabetes [8, 9].

The first step in the treatment of diabetes is to control serum glucose levels, which can be done in several ways, including the use of antidiabetic medications, physical activity, and insulin therapy [10, 11]. In one study, it was found that patients needed insulin therapy after 10 years of using antidiabetic medications. Insulin therapy has been shown to have some side effects, including lipohypertrophy and lipoatrophy [12].

Herbal medicine is a complementary method of diabetes treatment [13]. So far, many plants have been examined and used to prevent and treat diabetes. More than 1200 plants with antidiabetic properties have been described in the literature [14].

Some herbs have been tested and confirmed to have insulin sensitizing effects [15]. Some others have antihyperlipidemic [16] and antioxidant properties [17], which can be very useful in the treatment of chronic diseases [18]. Most medicinal plants contain alkaloids, flavonoids, carotenoids, glycosides, terpenoids, etc., which are often shown to have antidiabetic effects [19].

*Rheum ribes* (*R. ribes*) belongs to the plant family Polygonaceae [20]. It is used for medicinal purposes, such as diabetes [21], diarrhea [22], gastrointestinal bleeding [23], constipation [24], and its fresh forms are also consumed as vegetables [25]. It is widely used in Anatolia, Turkey, Iraq, and Iran [26]. In some studies, *R. ribes* was reported to improve renal failure [21], the glucose uptake [27], and diabetic nephropathy in experimental settings [28, 29]. The extract of *R. ribes* roots has shown significant glucose-lowering properties in the model of induced polycystic ovary syndrome in rabbits [30]. The cholesterol-lowering effect of both ethanolic and aqueous extracts of *R. ribes* in rabbits has also been reported [31].

The extract of *R. ribes* consists of numerous components such as tannins and anthracene derivatives [32]. The results of the digestion of *R. ribes* showed that the substances extracted from the plant include physcion, chrysophanol, aloe-emodin rhein, physcion-8-O-glucoside, sennoside A and rhaponticin [29]. Because of the profile of phenolic constituents of *R. ribes*, such as flavonoids, stilbenes, and anthraquinones, they are a great source of antioxidants [25].

The importance of *R. ribes*-derived extracts and *R. ribes* metabolites with diverse chemical backgrounds, such as anthraquinones and stilbenes, has been highlighted as potential modulators of human metabolism, including the prevention of cardiovascular diseases. In the present reports, the properties of *R. ribes* are described as follows: inhibition of enzymes of cholesterol and lipid metabolism, regulation of cellular energy metabolism, ability to inhibit proinflammatory signaling pathways and regulation of glucose and lipid homeostasis, raising the hypothesis that *R. ribes* may play a complementary role in the treatment of type 2 diabetes [33].

Some experimental studies have shown the effect of *R. ribes* on glycemic control in alloxan-treated diabetic rats [34–36]. Also, some clinical studies showed the anti-hyperglycemic and lipid-lowering effect of *R. ribes* in T2DM [37, 38]. However, few studies have been conducted to investigate the effect of *R. ribes* in diabetes. However, further studies in different diabetic populations and elimination of the limitations of previous studies are needed to explore its efficacy. Also, the effects of *R. ribes* on glycemic indices and apolipoprotein A-I (ApoA1), apolipoprotein B (ApoB), and ApoB/ApoA1 ratio of diabetic patients were not investigated in previous studies, and no comparison was made between the aqueous and ethanolic extract of *R. ribes* had been made in previous studies. Therefore, the current study aimed to investigate whether the aqueous and ethanolic extract of *R. ribes* improved glycemic status and apolipoproteins in patients with T2DM. To our knowledge, this is the first study to investigate the efficacy of two types of aqueous and ethanolic *R. ribes* extracts in diabetic patients.

## Method and materials

### Participants

In a randomised, placebo-controlled, double-blind clinical trial, T2DM patients aged 30–60 years old from Firoozgar Hospital in Tehran, Iran, were recruited for the current study. The Ethics Committee of Iran University of Medical Sciences approved the study protocol, and the study protocol was registered on website of the the Iranian Registry of Clinical Trials (identifier: IRCT201410142709N31, at the date of 11/12/2014). All methods were performed in accordance with relevant guidelines and regulations of Helsinki declaration. According to the study by Fallah Huseini et al., this study was designed with a power of 90% and a two sided a = 0.05 (type I error) to detect a 5% difference in serum glucose between the two group. Based on the SDs observed in the current study, the number of subjects who needed treatment to detect this difference was 16/group. Considering an expected dropout rate of 25%, we set a target for enrollment of 20 subjects [37].

### Inclusion and exclusion criteria

Inclusion criteria were as follows: Fasting blood glucose ≥ 126 mg/dl, age of participants between 30 and 60 years old and consistent medication schedule with metformin + glibenclamide. Diagnosis was done by an endocrinologist based on fasting blood glucose greater than 126 mg/dl.

Exclusion criteria were: Insulin treatment, sensitivity to *R. ribes* extract, pregnancy during the study, change in drug dosage during the study, intake of dietary supplements, smoking, alcoholism, consumption of green tea, and intake of other herbal medicines. History of known diseases such as liver, kidney, cardiovascular, thyroid and, gastrointestinal diseases, taking medications to lower blood lipids or blood pressure, taking corticosteroids, cyclosporine, nonsteroidal anti-inflammatory or immunosuppressive drugs, warfarin and antiepileptic drugs, pregnancy or lactation, and allergy to ragweed plants.

### Plant material and extract preparation

The rhubarb plant was obtained from Shahroud, Semnan, Iran. Subsequently, it was approved in the Department of Medicinal Plants, Faculty of Pharmaceutical Sciences, Shahid Beheshti University of Medical Sciences, Tehran, Iran. The voucher specimen was deposited in the herbarium of Iran University of Medical Sciences, Tehran, Iran (93–02-27–24,367). The study complies with relevant institutional, national, and international guidelines and legislation for plant ethics. For extraction, the whole plants were first washed and disinfected. Then they were completely dried by aeration and cut into small pieces. In the next step, equal amounts of the plant were used for ethanolic and aqueous extraction. The extraction process followed the soaking method. For this purpose, 100 g of dried rhubarb plant was first placed in a container, crushed and mixed with 1000 ml of distilled water and 1000 ml of absolute ethyl alcohol to produce an aqueous and alcoholic extract, respectively. Then, the contents of the container were spread on a flat paper for 4 h and placed on a bain-marie. The extract was obtained by evaporating the solvent, so that 8 g of extract were obtained from 100 g of the plant. The alcoholic extract was placed in ethanol alcohol in dark and sealed containers for 4 days. Then its content was filtered, the solid content was removed and the pure extract was prepared (1:10 w/v). To prepare the aqueous extract, the plant was immersed in water and boiled in a metal container, then brought to room temperature and placed on bain-marie after straining a filter paper. Finally, the extracts were converted into dry extracts and processed with corn starch to form granules and powder. Finally, the encapsulation step was performed.

### HPLC analysis of the phenolic/polyphenolic compounds of the aqueous and ethanolic extracts of *R. ribes*

100 mg of both extracts were dissolved in 5 ml of ethanol–water at a ratio of 80:20 v/v, then the mixtures were ultrasonicated at 25 °C for 25 min at 60% duty cycles. After this step, the mixtures were centrifuged at 7500 rpm for 15 min. The supernatant of the extracts was treated with charcoal to remove pigments removal and then evaporated in vacuo. The dried extracts were mixed with 1 ml of HPLC methanol by shaking, and the suspensions were passed through a 2.5 μm filter and stored at 4 °C until analysis. The extracts were injected into the HPLC system under optimal conditions. HPLC was performed using a Shimadzu 10 AV-LC, and peaks were detected and monitored using a UV–Vis 10AV-SPD spectrophotometer (Shimadzu, Kyoto, Japan). In addition, peaks found in the extracts were detected by comparison with the suspensions of the standard materials.

### Study design

After sample selection, a general information questionnaire was completed for each patient. Body weight was measured for all patients with a scale (Seca, Hamburg, Germany), without shoes and in light clothing. Body height was measured with a mounted tape measure and without shoes. BMI was calculated as weight in kilograms divided by height in meters squared.

Information on daily energy and macronutrient intake was obtained by a three-day dietary recall that included two regular days and one weekend day. Average three-days dietary data were analyzed using Nutritionist 4 software for all patients at baseline and at the end of sixth weeks of the study (First Databank Inc., Hearst Corp., San Bruno, CA).

The patients were stratified for sex, weight and age before the intervention, and then were randomly assigned (balanced-block method) into three groups, each group consisted of 20 patients, as the aqueous extract group (AG, *n* = 20), the ethanolic extract group (EG, *n* = 20) and placebo (control) group (PG, *n* = 20). Patients in AG and EG groups received 3 *R. ribes* capsules daily for 6 weeks. Each capsule contained 450 mg of the *R. ribes* extract (3 × 450 mg daily). The placebo (control) group (PG, *n* = 20) received 3 placebo capsules (contained starch), similar in appearance, shape, and color to the capsules of AG and EG. The capsules of *R. ribes* were prepared by the Department of Medicinal Plants, Faculty of Pharmacy, Shahid Beheshti University of Medical Sciences, Tehran, Iran.

Patients were recruited by simple random sampling. Enrolled patients were assigned to three groups based on a balanced block randomization list (using Epi Info™ software). Allocation was also based on sequentially numbered opaque pockets (A: treatment group, and B: treatment group, and C: control group), and prescribed medications were packaged in the same containers labeled by A, B, and C, respectively. The patients and researcher were blinded about the content.

Subjects’ compliance with the study protocol was assessed once a week by telephone interviews and every two weeks by counting the number of capsules returned.

### Biochemical assessment

Fasting blood samples (10 ml) were drawn from the brachial artery of all participants after 12 h of fasting at baseline and end of sixth week of the intervention. Serum samples were immediately centrifuged at 10,000 rpm for 20 min (Sigma, UK) to separate the serum. Then, the serum samples were stored at -70 °C in the reference laboratory of Iran University of Medical Sciences before analysis. Serum glucose, ApoB, ApoA1 and insulin were measured by the standard enzymatic method of glucose oxidase (Pars Azmoon, Tehran, Iran), immunoturbidimetry method (Pars Azmoon, Tehran, Iran), immunoturbidimetry method (Pars Azmoon, Tehran, Iran), and ELISA method (Pars Azmoon, Tehran, Iran), respectively. Insulin resistance and β-cell dysfunction were determined using the Homeostasis Model Assessment (HOMA-IR) and Homeostasis Model Assessment-beta (HOMA-B), respectively and according to the following formula: [glucose (nmol/L) x insulin (microU/L) /22.5] and [20 × insulin (μIU/ml) /glucose (mmol/ml) -3.5] %, respectively.

### Statistical methods

Data obtained from the tests and interviews were entered into SPSS software, version 22, and then subjected to statistical analysis. All variables were reported on the basis of mean and standard deviation. The Kolmogorov–Smirnov test was used to determine the distribution of data. Paired t-test was used for comparison of normally distributed variables within group before and after the study. The ANOVA was used for comparison of the means of three groups. After ANOVA was run and it found significant results overall, then Tukey’s Honest Significant Difference test, a post-hoc test based on the studentized range distribution, was used to find out which specific groups’ means (compared with each other) are different. The ANCOVA test was used to adjust the effect of variables such as age and baseline level of variables. The statistical test of multiple linear regression was used to show the association of *R. ribes* supplementation with insulin, HOMA-IR, HOMA-B, ApoA1, and ApoB/ApoA1 ratio adjusting the baseline values of age and weight of patients. In addition, the statistical method of analysis of variance with repeated measures was used to verify the information of patients’ dietary intake. The confidence level for all variables was set at 95%. The level of statistical significance was *P* < 0.05.

## Results

HPLC analysis showed that the ethanolic and aqueous extracts of *R. ribes* contained some phytochemicals and polyphenols. The phenolic compounds of the ethanolic extract were higher compared to those of the aqueous extract (Table 1).

**Table 1:** Phenolic/polyphenolic content of ethanolic and aqueous extracts of R. ribes

Phenolic/polyphenolic compounds	Ethanolic extract (μg/ml)	Aqueous extract (μg/ml)
Emodin	2021.85	1187.23
Aloe emodin	1045.36	317.41
Physcion	1094.52	1588.55
Chrysophanol	2199.49	649.83
Rhein	409.28	156.89
Rutin	229.19	183.17
Chlorogenic acid	557.17	58.91
Tannic acid	115.65	49.32
Kaempferol	122.74	111.54
Gallic acid	291.29	166.10

**Fig. 1:**
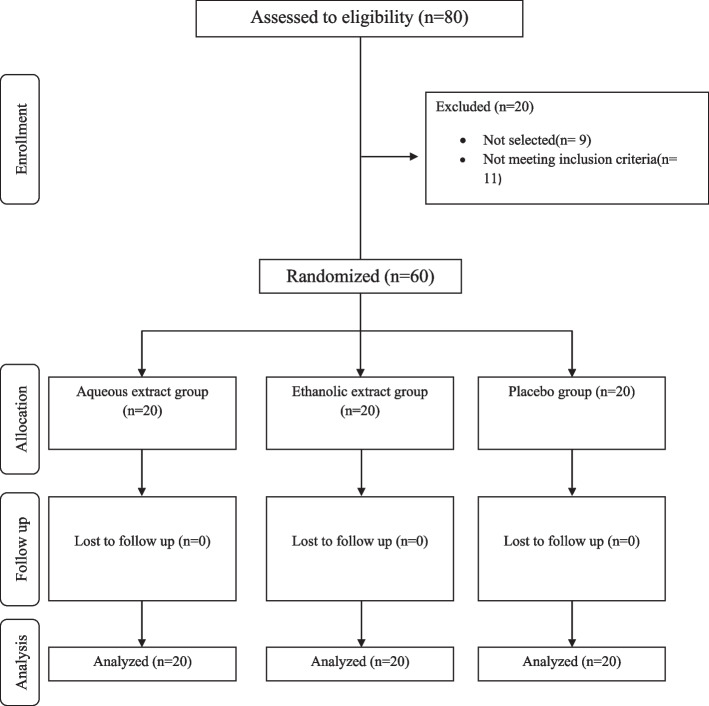
Summary of patient flow diagram

Of the 60 study participants, all were present until the end of the study, and no adverse effects of the ethanolic and aqueous rhubarb extracts were reported. The mean age of the study participants was 54.08 ± 5.39 years (Fig. 1 and Table 2).

**Table 2:** General characteristics of participants

Variable	AG (*n* = 20)	EG (*n* = 20)	PG (*n* = 20)	*p*-value^a^
Number, men/women	20, 12/8	20, 13/7	20, 10/10	0.15
Age (years)	53.35 ± 5.58	53.70 ± 4.11	55.20 ± 4.44	0.94
Weight (Kg)	76.95 ± 4.07	76.25 ± 7.36	73.80 ± 26.73	0.36
BMI (Kg/m^2^)	27.14 ± 2.72	25.51 ± 1.90	26.73 ± 2.11	0.29

*AG* Aqueous extract group, *EG* Ethanolic extract group, *PG* Placebo group

^a^Obtained from ANOVA test

Subjects had no significant differences in baseline characteristics (Table 2). At the end of the study, the mean BMI of AG, EG, and PG were 26.6 ± 2.2, 25.24 ± 1.7, and 26.5 ± 2.0 kg/m^2^, respectively, and these values did not show significant changes within groups (*P* = 0.80, *P* = 0.14, and *P* = 0.40, respectively) and between groups (*P* = 0.06). There were also no significant changes in the average dietary intake of the participants among the groups during the study (Table 3).

**Table 3:** Dietary intakes of study participants throughout the study

Variable	AG (*n* = 20)				EG (*n* = 20)				PG (*n* = 20)				*p*-value^b^
	Week 0	Week 3	Week 6	*p*-value^a^	Week 0	Week 3	Week 6	*p*-value^a^	Week 0	Week 3	Week 6	*p*-value^a^	
Energy (Kcal)	2214.00 ± 426.61	2202.00 ± 389.31	2182.00 ± 408.01	0.06	2108.50 ± 381.81	2130.50 ± 298.05	2127.61 ± 314.21	0.15	2134.50 ± 293.73	2109.50 ± 269.41	2124.31 ± 124.58	0.09	0.18
Carbohydrate (g/d)	317.15 ± 46.33	303.40 ± 37.80	323.00 ± 29.70	0.86	297.85 ± 44.93	298.50 ± 45.38	302.54 ± 39.56	0.57	300.00 ± 41.31	309.60 ± 40.95	325.18 ± 57.69	0.74	0.28
Protein (g/d)	72.30 ± 34.76	71.65 ± 28.98	69.95 ± 27.12	0.38	65.35 ± 29.68	65.20 ± 28.04	64.91 ± 31.10	0.20	60.85 ± 18.64	59.40 ± 18.27	61.25 ± 17.20	0.06	0.13
Total fat (g/d)	81.60 ± 17.99	78.25 ± 14.25	85.81 ± 18.17	0.45	78.60 ± 14.60	79.45 ± 9.19	79.14 ± 10.09	0.85	83.85 ± 9.10	81.30 ± 14.25	84.00 ± 10.81	0.14	0.61
SAFA (g/d)	22.98 ± 6.34	19.34 ± 3.91	21.47 ± 2.11	0.34	20.05 ± 5.06	19.88 ± 5.11	19.93 ± 6.47	0.75	20.00 ± 4.12	22.29 ± 5.44	21.94 ± 4.85	0.17	0.82
MUFA (g/d)	19.75 ± 4.33	18.33 ± 4.57	19.81 ± 5.15	0.98	17.73 ± 3.63	15.65 ± 1.65	16.76 ± 2.95	0.45	16.08 ± 2.38	18.72 ± 4.29	18.66 ± 4.62	0.25	0.74
PUFA (g/d)	11.10 ± 2.42	11.11 ± 1.34	11.08 ± 2.07	0.27	11.42 ± 2.06	11.27 ± 1.63	11.64 ± 1.80	0.06	11.39 ± 1.67	11.57 ± 2.23	11.44 ± 2.17	0.09	0.19
Vitamin A (mg)	415.25 ± 66.74	386.15 ± 51.46	367.27 ± 45.34	0.07	388.65 ± 67.92	417.45 ± 61.85	397.27 ± 55.24	0.52	390.85 ± 48.69	397.85 ± 62.35	400.21 ± 51.47	0.096	0.36
Vitamin E (mg)	4.41 ± 0.89	4.89 ± 0.80	4.47 ± 0.99	0.63	4.61 ± 0.93	4.91 ± 0.81	4.68 ± 0.97	0.85	4.89 ± 0.80	4.67 ± 0.93	4.80 ± 0.12	0.08	0.91
Vitamin D (μg)	5.15 ± 0.51	5.00 ± 0.62	5.08 ± 0.81	0.58	4.86 ± 0.72	5.08 ± 0.62	5.05 ± 0.24	0.95	4.81 ± 0.70	4.82 ± 0.67	4.20 ± 0.90	0.25	0.82
Vitamin C (mg)	17.75 ± 3.70	16.72 ± 3.17	17.28 ± 1.18	0.45	16.83 ± 3.33	17.59 ± 3.14	17.04 ± 2.67	0.14	16.84 ± 3.03	16.19 ± 2.76	16.96 ± 2.55	0.076	0.06
Calcium (mg)	1056.30 ± 167.68	1019.00 ± 127.36	1087.01 ± 133.36	0.08	948.35 ± 240.95	901.95 ± 177.93	901.95 ± 185.76	0.28	892.20 ± 168.80	917.50 ± 174.85	908.63 ± 185.25	0.59	0.98
Iron (mg)	13.56 ± 2.69	10.90 ± 2.10	10.74 ± 10.54	0.06	13.01 ± 2.60	13.32 ± 2.83	13.05 ± 2.21	0.92	10.85 ± 2.38	12.60 ± 2.49	11.91 ± 1.24	0.25	0.76
Selenium (μg)	0.05 ± 0.01	0.04 ± 0.01	0.04 ± 0.18	0.09	0.04 ± 0.01	0.04 ± 0.00	0.04 ± 0.00	0.63	0.04 ± 0.00	0.04 ± 0.00	0.04 ± 0.01	0.39	0.49
Zinc (µg)	7.78 ± 1.15	7.31 ± 1.09	7.97 ± 1.85	0.12	6.76 ± 1.62	7.26 ± 1.02	7.04 ± 1.51	0.45	6.01 ± 1.00	6.06 ± 1.09	6.04 ± 1.02	0.81	0.09
Fiber (g/d)	13.70 ± 2.60	14.37 ± 2.50	1.98 ± 1.84	0.74	14.19 ± 3.64	14.48 ± 3.23	14.20 ± 3.17	0.25	15.19 ± 2.80	15.17 ± 3.18	15.22 ± 3.84	0.79	0.70

Data are means ± standard deviations

*AG* Aqueous extract group, *EG* Ethanolic extract group, *PG* Placebo group, *SFA* Saturated fatty acid, *PUFA* Polyunsaturated fatty acid, *MUFA* Monounsaturated fatty acid

^a^*p*-value for repeated measures ANOVA performed to assess variations in dietary intakes across periods

^b^Obtained from ANOVA test on dietary intakes at week 6

The results showed that the glycemic indices within each group of AG and EG were significantly decreased at the end of study compare to baseline values, including insulin (*P* = 0.003 and *P* = 0.001, respectively), HOMA-IR (*P* = 0.01 and *P* = 0.001, respectively), and HOMA-B (*P* = 0.002 and *P* = 0.001, respectively), but there were no significant changes in serum glucose levels within each group during the study (*p* > 0.05) (Table 4).

**Table 4:** Effect of aqueous and ethanolic extracts of R. ribes intake on glycemic indices and apoproteins

Variable	AG (*n* = 20)			EG (*n* = 20)			PG (*n* = 20)			*P*-value^b^
	Before	After	*P*-value^a^	Before	After	*P*-value^a^	Before	After	*P*-value^a^	
glucose (mg/dL)	145.75 ± 29.95	140.85 ± 21.85	0.09	139.43 ± 26.82	136.56 ± 25.39	0.11	131.34 ± 23.67	128.65 ± 22.82	0.11	0.78
Insulin (μIU/ml)	7.04 ± 5.14	6.24 ± 4.35	0.003	8.09 ± 4.14	6.63 ± 3.59	0.001	8.13 ± 4.32	8.45 ± 3.97	0.13	0.04*
HOMA-IR	2.17 ± 1.50	1.85 ± 1.30	0.01	2.09 ± 0.97	2.03 ± 1.14	0.001	2.20 ± 1.31	2.22 ± 1.19	0.11	0.03*
HOMA-B	1.36 ± 0.91	1.05 ± 0.74	0.002	1.40 ± 0.67	1.17 ± 0.70	0.001	1.21 ± 0.59	1.27 ± 0.68	0.81	0.01*
ApoA1 (mg/dl)	147.40 ± 21.53	151.90 ± 21.53	0.08	115.05 ± 36.36	122.40 ± 23.12	0.05	154.05 ± 28.56	142.65 ± 24.84	0.09	0.13
ApoB (mg/dl)	92.95 ± 29.39	85.60 ± 20.30	0.006	89.70 ± 24.42	88.55 ± 14.53	0.03	89.91 ± 17.77	93.50 ± 19.88	0.35	0.02*
ApoB/ApoA1 ratio	0.58 ± 0.14	0.56 ± 0.14	0.016	0.80 ± 0.14	0.74 ± 0.17	0.04	0.59 ± 0.13	0.58 ± 0.09	0.08	0.03*

All values are means ± SD

*AG* Aqueous extract group, *EG* Ethanolic extract group, *PG* Placebo group, *HOMA-IR* Homeostasis model of assessment-insulin resistance, *HOMAB* Homeostatic model assessment-Beta cell function, *ApoB* Apolipoprotein-B, *ApoA1* Apolipoprotein AI

^a^Obtained from paired T-Test

^b^Obtained from ANOVA test

^*^Significant difference in AG and EG as compared to PG according to ANOVA/Tukey

As for apolipoproteins, the results in all groups showed that ApoB was significantly decreased at the end of the study compared to the initial levels in AG and also in EG (*P* = 0.006 and *P* = 0.03, respectively). The ApoB/ApoA1 ratio also decreased significantly in both AG and EG groups at the end of the study compared with the initial values (*P* = 0.016 and *P* = 0.04, respectively). The mean serum levels of ApoA1 were increased at the end of the study compared to baseline levels in both AG (*P* = 0.08) and EG (*P* = 0.05) groups (Table 4).

At the end of the study, the results of ANOVA/Tukey showed that insulin, HOMA-B, HOMA-IR, ApoB and ApoB/ApoA1 ratio had significant decrease (*P* = 0.04, *P* = 0.01, *P* = 0.03, *P* = 0.02 and *P* = 0.03, respectively) in AG and EG compared to PG, but there were no significant differences between AG and EG (Table 4).

The serum levels of these variables after the intervention also had statistically significant differences among the three groups, as shown by the ANCOVA test, excluding the confounding effect of insulin, glucose, ApoA1, ApoB, and age before the intervention (*P* < 0.001) (Table 5). The statistical test of multiple linear regression also showed that serum levels of insulin, HOMA-IR, HOMA-B, ApoA1, and the ratio of ApoA1 to ApoB were significantly associated with *R. ribes* supplementation after adjusting for baseline levels of age and also weight (Table 6).

**Table 5:** Adjusted changes in metabolic variables in study participants

Variable	AG (*n* = 20)	EG (*n* = 20)	PG (*n* = 20)	*P*-value^a^
Insulin (μIU/ml)				
Model 1^b^	-0.82 ± 0.98	-1.48 ± 0.48	0.41 ± 0.85	0.03
Model 2^c^	-0.84 ± 0.98	-1.48 ± 0.48	0.40 ± 0.85	0.03
HOMA-IR				
Model 1	-0.35 ± 0.14	0.08 ± 0.09	0.02 ± 014	0.03
Model 2	-0.36 ± 0.14	0.09 ± 0.09	0.05 ± 013	0.03
HOMA-B				
Model 1	-0.38 ± 0.23	-0.26 ± 0.08	-0.05 ± 0.12	0.01
Model 2	-0.39 ± 0.23	-0.25 ± 0.08	-0.04 ± 0.11	0.01
ApoB (mg/dl)				
Model 1	-12.19 ± 9.74	-1.88 ± 10.21	-3.98 ± 3.45	0.03
Model 2	-12.11 ± 9.74	-1.82 ± 10.21	-3.90 ± 3.46	0.03
ApoB/ApoA1				
Model 1	-0.02 ± 0.01	0.71 ± 0.02	-0.01 ± 0.03	0.04
Model 2	-0.04 ± 0.01	0.75 ± 0.02	-0.02 ± 0.03	0.04

All values are means ± standard errors

*AG* Aqueous extract group, *EG* Ethanolic extract group, *PG* Placebo group, *HOMA-IR* Homeostasis model of assessment-insulin resistance, *HOMAB* Homeostatic model assessment-Beta cell function, *ApoB* Apolipoprotein-B, *ApoA1* Apolipoprotein AI

^a^Obtained from ANCOVA

^b^Adjusted for baseline values

^c^Further adjusted for Model 1 + age

**Table 6:** Multiple linear regression beta-coefficients (95% Confidence interval) describing the association of study variables with R. ribes consumption

Variable^a^	beta-coefficient (95% CI) AG (*n* = 20)	beta-coefficient (95% CI) EG (*n* = 20)	beta-coefficient (95% CI) PG (*n* = 20)	*P*-value
Insulin (μIU/ml)	-0.18 (-0.38; -0.11)	-0.24 (-0.45; -0.14)	0.12 (-0.03; 0.19)	0.02
HOMA-IR	-0.12 (-0.29; -0.03)	0.04 (-0.02; 0.10)	0.01 (-0.01; 0.04)	0.02
HOMA-B	-0.13 (-0.27; -0.08)	-0.12 (-0.26; -0.09)	-0.08 (-0.15; 0.03)	0.01
ApoB (mg/dl)	-0.05 (-0.09; -0.02)	-0.7 (-0.17; -0.03)	-0.18 (-0.38; -0.11)	0.02
ApoB/ApoA1	-0.005 (-0.009; -0.001)	0.004 (0.001; 0.007)	0.006 (-0.002; 0.009)	0.04

*AG* Aqueous extract group, *EG* Ethanolic extract group, *PG* Placebo group, *HOMA-IR* Homeostasis model of assessment-insulin resistance, *HOMAB* Homeostatic model assessment-Beta cell function, *ApoB* Apoprotein-B, *ApoA1* Apoprotein AI

^a^Adjusted for baseline values age and baseline weight

## Discussion

In the present study, daily supplementation of aqueous and ethanolic *R. ribes* extract for 6 weeks was led to significant changes in insulin, HOMA-B, HOMA-IR, ApoB and ApoB/ApoA1 ratio in patients with T2DM compared to control group, but serum levels of ApoA1 was not significantly increased in these groups compare to control group. The mean dietary intake among the groups did not show significant differences, so dietary intake could not be a confounding factor for the results of the study.

Hamzeh et al. showed in their study that daily oral intake of a hydroalcoholic *R. ribes* extract (150 mg/kg) for a period of 4 weeks resulted in a significant decrease in serum glucose levels in alloxan-monohydrate induced diabetic rats [28].

Ozbek et al. investigated the efficacy of *R. ribes* root extract in alloxan-treated diabetic rats, and observed it’s hypoglycemic effect [34]. In another study, Radhika et al. showed a significant decrease in plasma glucose levels in alloxan-treated diabetic rats in a study with 250 mg ethanolic extract of *R. ribes* per kilogram of body weight [35]. Rafaat et al. also showed blood glucose-lowering effects in alloxan-treated diabetic rats in their study of the phytotherapeutic effects of *R. ribes* [39]. In another study, Kasabri et al. reported the blood glucose-lowering effect of *R. ribes* in a dose-dependent manner in diabetic rats [36].

In the study by Chen et al., several stilbene compounds, including rhaponticin, isolated from rhubarb rhizome extract, and the effects of rhaponticin on glucose and lipid metabolism as well as liver and heart function were investigated in a rat model of T2DM. Rhubarb supplementation significantly decreased serum levels of glucose and insulin, and this supplementation resulted in higher insulin sensitivity in diabetic mice [40].

In an experimental study by Naqishbandi et al., the serum glucose levels of healthy mice decreased after administration of a single dose of *R. ribes* extract [41]. Studies have shown that serum levels of glycemic indices and lipid profile are altered in patients with T2DM [38]. In another study conducted by Fallah Huseini et al*.* the evaluation of the effect of *R. ribes* supplementation in hyperlipidemic patients with T2DM showed a decrease in the levels of glucose, LDL and total cholesterol levels [42]. The results of these studies were consistent with our results regarding the effect of *R. ribes* effect on insulin sensitivity, but our study was inconsistent with other studies due to no effect of *R. ribes* on serum glucose. However, in the study by Fallah Huseini et al., the participants were hyperlipidemic (nondiabetic) and had different blood glucose levels than our study population, and the duration of the intervention and the dose of the extract were also different from those in our study, which could be the possible reasons for these inconsistencies. Insulin resistance, HOMA-IR, and HOMA-B were significantly decreased in AG and EG compared with control group(PG), suggesting that both the alcoholic and aqueous extracts of *R. ribes* had beneficial effects on insulin resistance and ß-cell function in diabetic patients.

One study indicated that rhubarb polyphenols increased insulin-stimulated glucose uptake as well as pioglitazone in adipocytes, suggesting that the active metabolites of rhubarb may enhance insulin action via peroxisome proliferator-activated receptor-γ (PPAR-γ) activation [43]. Rhubarb also acts as a pro-drug and has an inhibitory effect on glucose uptake in the small intestine and glucose reabsorption in the renal tubular [44]. Rhubarb can activate PPAR-γ coactivator-1α (PGC-1α), 5' AMP-activated protein kinase (AMPK), and sirtuin 1 (SIRT1), which have been proposed as potential targets for the development of therapeutic approaches of T2DM [45, 46].

In this study, we investigated the effect of *R. ribes* on apolipoproteins and found that rhubarb and its active metabolites can decrease serum levels of ApoB. ApoA1 is the major apolipoprotein of HDL and plays a critical role in the transfer of excess cholesterol from tissues to the liver. Plasma concentrations of atherogenic lipoprotein particles, as measured by ApoB, are more informative for the development of coronary heart disease than cholesterol, as measured by non-high-density lipoprotein (non-HDL) cholesterol and, in particular, the ApoB/ApoA1 ratio, which is a more important predictor of cardiovascular disease compared with LDL/HDL cholesterol. Patients with T2DM have higher levels of small dense LDL (ApoB) compared with healthy individuals, which increases the risk of cardiovascular disease (CVD) in T2DM. The significant decrease in Apo B-100 at the end of the study in the AG and EG group compared to PG indicates a decrease in the number of LDL and VLDL particles. This is expected to contribute to a reduction in atherogenic risk in patients with type 2 diabetes [47, 48].

These apolipoproteins are also a useful indicator for lipid-lowering therapies [49]. Insulin resistance leads to the development of hyperinsulinemia, which can also burn pancreatic beta cells and lead to T2DM. In addition, increased lipid flux along with insulin resistance leads to overproduction of atherogenic ApoB containing apolipoproteins (VLDL, LDL, and moderate lipoprotein). High plasma levels of ApoB lead to white adipose tissue dysfunction and type 2 diabetes. White adipose tissue dysfunction may increase the risk of T2DM [50]. ApoB is a particularly sensitive indicator of changes in glycemic control, so it is possible that tight glycemic control (reduction of insulin resistance in our study) may have ‘antiatherogenic’ effects by lowering ApoB levels (which were also lowered in our study). Since ApoB is directly related to T2DM risk and *R. ribes* had ameliorative effects on both glycemic indices and apolipoproteins in the present study, it is likely to have a protective effect on type 2 diabetes [51, 52].

Previously, the active constituents of *R. ribes* were shown to have an antioxidant effect [53], and the improvement in insulin sensitivity and lipid profile may be due to the antioxidant effect of *R. ribes,* which may affect insulin secretory cell function and lipid oxidation. In the study by Matsuda et al., some active components of *R. ribes* extract showed antioxidant properties, including emodin and its derivatives [53]. In our extract, emodin and its derivatives have the highest concentration among the active components, and it seems that the antioxidant properties showing antidiabetic effects are related to emodin and its derivatives.

To our knowledge, this study was the first study to compare aqueous and alcoholic extracts of *R. ribes* with each other and with a control group in diabetic patients. However, this study has some limitations, such as the short intervention period (6 weeks), and it would have been better if the intervention has continued up to 12 weeks, but this was not possible because the subjects could not come to the study center due to time constraints and also compliance would have been limited if the authors had extended the period. In addition, the hemoglobin A1c level, as a better predictor of glycemic status which indicate glycemic indices status in the last two months., was not measured. There was a lack of comparison of different doses of extracts that could show the minimum effective doses of extracts on the studied variables. The present study was conducted with 60 patients in three groups, including an aqueous extract, an alcoholic extract, and a control group, and it was better to study more patients and different doses of the two extracts. In addition, we used a 24-h recall, a memory-based method, for 3 days at beginning and also end of study to evaluate food intake, in which recall errors may be occurred. Further studies with more participants of T2DM and longer time duration are needed to evaluate the effects of different doses of the of *R. ribes* on glycemic status and apolipoproteins.

## Conclusion

Both the alcoholic and aqueous extracts of *R. ribes* had beneficial effects on insulin resistance and apolipoprotein levels in patients with T2DM, but it seems that these two extracts had similar potency for these effects. However, due to rare scientific reports in this field, further studies are needed.

## Data Availability

The datasets generated and/or analyzed during the current study are not publicly available due to disagreement of some authors of the manuscript but are available from the corresponding author on reasonable request.

## Abbreviations

T2DM: Type 2 diabetes mellitus
BMI: Body mass index
HbA1c: Hemoglobin A1c
AG: *Rheum ribes* Aqueous extract
EG: *Rheum ribes* Ethanolic extract
PG: Placebo
HOMA-IR and HOMA-B: The homeostatic model assessment of β-cell dysfunction
ApoA1: Apolipoprotein A-I
ApoB: Apolipoprotein B
PPAR-γ: Peroxisome proliferator-activated receptor-γ
PGC-1α: PPAR-γ coactivator-1α
AMPK: AMP-activated protein kinase
SIRT1: Sirtuin 1
non-HDL: Non-high density lipoprotein
CVD: Cardiovascular disease
